# PD-1/PD-L1 Inhibitors and Chemotherapy Synergy: Impact on Drug Resistance and PD-L1 Expression in Breast Cancer-Immune Cell Co-Cultures

**DOI:** 10.3390/ijms26146876

**Published:** 2025-07-17

**Authors:** Güneş Özen Eroğlu, Ayşe Erol Bozkurt, İlhan Yaylım, Dürdane Serap Kuruca

**Affiliations:** 1 Department of Molecular Medicine, Aziz Sancar Institute of Experimental Medicine, Istanbul University, Istanbul 34093, Turkey; ilhanyaylim@gmail.com; 2 Institute of Graduate Studies in Health Science, Istanbul University, Istanbul 34126, Turkey; 3 Department of Medical Biology, Faculty of Medicine, Istanbul University, Istanbul 34390, Turkey; ayse.erol@istanbul.edu.tr; 4 Department of Physiology, Faculty of Medicine, Istanbul Atlas University, Istanbul 34408, Turkey; serap.kuruca@atlas.edu.tr

**Keywords:** breast cancer, PD-L1, immunotherapy, chemotherapy, drug resistance

## Abstract

Breast cancer is the most frequently diagnosed cancer among women. In recent years, immunotherapy, a key targeted treatment strategy, has gained prominence in the management of this disease. Immune cells within the tumor microenvironment can significantly affect treatment outcomes. Among immunotherapeutic approaches, or programmed death protein 1(PD-1) and programmed death-ligand 1(PD-L1)-targeted therapies are increasingly recognized for their role in modulating cancer–immune system interactions. This study investigated the impact of PD-1/PD-L1 pathway inhibition on the expression of drug resistance-related proteins in an in vitro breast cancer model incorporating immune cells. MDA-MB-231 and MCF-7 cell lines were used as breast cancer cells, while THP-1 and Jurkat cells represented monocytes and lymphocytes, respectively. The effects of paclitaxel (PTX), doxorubicin (Dox), and PD-1/PD-L1 inhibitors (BMS-1166 and Human PD-L1 Inhibitor IV (PI4)) on cell viability were evaluated using an MTT assay, and the IC_50_ values were determined. Flow cytometry was used to analyze PD-1/PD-L1 expression and the drug resistance proteins ABCG2 (ATP-binding cassette sub-family G member 2, breast cancer resistance protein), MDR-1 (multidrug resistance protein 1), and MRP-1 (multidrug resistance-associated protein 1) across co-culture models. Based on the results, Dox reduced PD-L1 expression in all groups except for MDA-MB-231:THP-1, while generally lowering drug resistance protein levels, except in MDA-MB-231:Jurkat. BMS-1166 significantly decreased cell viability and enhanced chemotherapy-induced cytotoxicity. Interestingly, in the MDA-MB-231:Jurkat co-culture, both inhibitors reduced PD-L1 but increased drug resistance protein expression. Paclitaxel’s effect on PD-L1 varied depending on the immune context. These findings highlight that PD-1/PD-L1 inhibitors and chemotherapeutic agents differentially affect PD-L1 and drug resistance-related protein expression depending on the immune cell composition within the tumor microenvironment.

## 1. Introduction

Despite extensive research efforts and advancements in therapeutic strategies, breast cancer remains the most commonly diagnosed malignancy among women worldwide, with approximately 2.3 million new cases and around 670,000 deaths reported globally in 2022, according to GLOBOCAN data [1]. For many years, cytotoxic chemotherapeutic agents represented the primary treatment option for breast cancer (BC) patients. Over time, a range of targeted therapeutic strategies has emerged to address the complexity of BC, which is marked by distinct molecular subtypes and clinical stages [2]. Among these strategies, cytotoxic agents such as Dox and PTX remain commonly administered, particularly in cases of metastatic breast cancer [3]. Immunotherapeutic approaches that target a range of molecular pathways have become integral components of current strategies. Cancer immunotherapies that target the immunosuppressive checkpoint receptors cytotoxic T-lymphocyte-associated protein 4 (CTLA4) or PD-1 and its ligand PD-L1, have changed the landscape of anticancer immunotherapy [4].

Tumor cells employ various immunoregulatory mechanisms to suppress antitumor immune responses. One of these mechanisms involves the overexpression of inhibitory receptors, also known as immune checkpoints. PD-1, also known as CD279, is an inhibitory molecule that regulates both adaptive and innate immune responses. It is expressed on activated T cells, natural killer cells, B lymphocytes, macrophages, dendritic cells, and monocytes [5].

Physiologically, PD-1 has two known ligands: PD-L1 and PD-L2. It has been reported that when cancer cells are targeted by the immune system, the expression of PD-L1 and PD-L2 increases, leading to T cell suppression and immune evasion [6]. PD-1 and PD-L1 expression have been demonstrated in a wide range of malignancies, including bladder, small cell lung, ovarian, and renal cancers [7]. PD-L1, functioning as a negative regulator of the immune response, is typically associated with “hot” tumor microenvironments characterized by active immune infiltration [8].

In line with the impact of PD-1/PD-L1 interaction on the tumor microenvironment and immune evasion, high PD-L1 expression in breast cancer has also been reported to correlate positively with poor prognosis. Particularly in triple-negative breast cancer (TNBC), a subtype known for its high metastatic potential, PD-L1 upregulation has been linked to aggressive characteristics, such as advanced histological grade, the absence of estrogen receptor (ER) expression, and increased infiltration by regulatory T cells (Tregs) [9,10].

The tumor microenvironment, particularly immune cell interactions, plays a crucial role in modulating therapeutic responses. Jurkat cells represent an immortalized human T lymphocyte line widely employed in research on acute T cell leukemia and T cell signaling mechanisms [11]. The THP-1 monocyte cell line was employed as a model to characterize monocyte behavior and to simulate the in vivo microenvironment of solid breast tumors [12]. In our study, breast cancer cells (MDA-MB-231 and MCF-7 cell lines) were co-cultured with immune cells (Jurkat and THP-1 cell lines) to mimic the breast tumor microenvironment, particularly focusing on the PD-1/PD-L1 receptor–ligand interaction.

Targeting immune checkpoint proteins using monoclonal antibodies has marked a major breakthrough in cancer therapy [13]. In contrast to antibody-based therapeutics, which are characterized by prolonged production timelines and high manufacturing costs, small molecule inhibitors, particularly peptide-based inhibitors, have emerged as promising candidates in anticancer therapy due to their high target specificity, short plasma half-life, low immunogenicity, and favorable pharmacokinetic properties, including efficient cellular membrane permeability and enhanced tumor tissue penetration resulting from their low molecular weight [14,15,16].

BMS-1166 is a specifically designed PD-1/PD-L1 interaction inhibitor that alleviates PD-L1-mediated suppression of T cell receptor-dependent T lymphocyte activation, thereby enhancing the antitumor immune response and exerting anticancer effects [17,18,19].

Human PD-L1 inhibitor IV also a rationally designed peptide-based inhibitor, exhibits the potential to block PD-1/PD-L1 interaction by binding directly to PD-1 [20].

Numerous studies have established that Dox and PTX modulate PD-L1 expression and contribute to drug resistance [21,22].

Several studies have demonstrated that PD-1/PD-L1 interaction contributes to chemoresistance in breast cancer by upregulating the expression of MDR-1, implying a potential PD-1/PD-L1-mediated mechanism underlying treatment resistance [23].

PTX, a taxane-based chemotherapeutic, and Dox, an anthracycline derivative, are widely used in breast cancer therapy due to their potent antitumor effects. However, both agents are also associated with the development of drug resistance. In breast cancer and various other malignancies, PTX and Dox have been reported to enhance the expression of major efflux transporters involved in drug resistance, including P-gp/MDR-1, MRP-1, and ABCG2/BCRP [24,25,26].

It is well established that the expression of multiple drug resistance proteins tends to increase both at the onset and throughout the course of cancer treatment. As multidrug resistance remains a major barrier to successful therapy and is closely linked to cancer relapse, elucidating how chemotherapeutic and immunotherapeutic strategies modulate resistance pathways is crucial for optimizing treatment efficacy [27].

Currently, there are no studies investigating the potential of BMS-1166 and PI4 inhibitors to modulate drug resistance. In this study, the effects of these inhibitors on the expression levels of PD-1, PD-L1, and drug resistance-related proteins, including ABCG2, MDR-1, and MRP-1, were evaluated across breast cancer cell lines within distinct immune microenvironment contexts.

## 2. Results

### 2.1. Co-Culture Model Optimization

**Figure 1:**
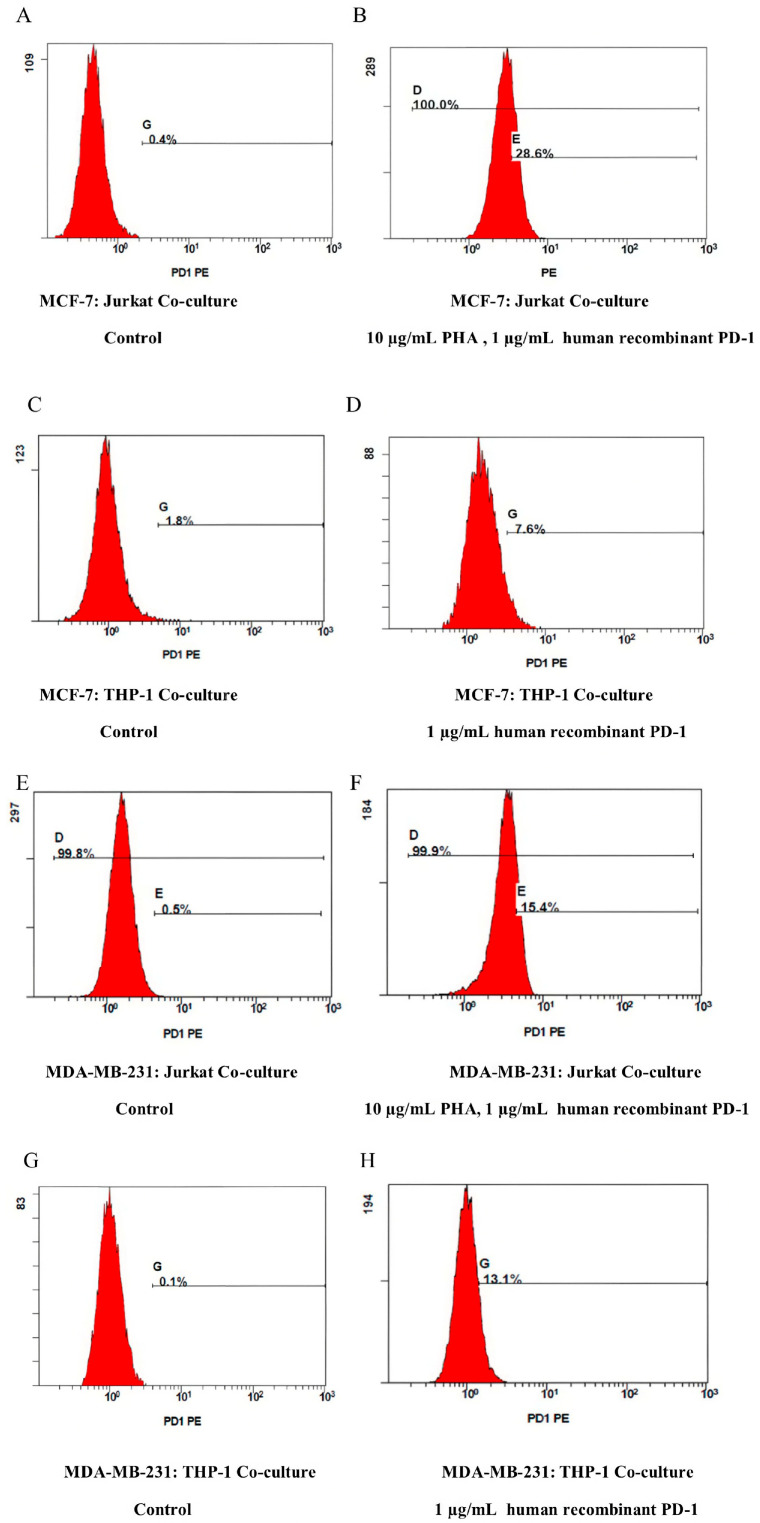
Alterations in PD-1 expression in co-cultured cells, analyzed via flow cytometry upon stimulation with PHA-M and human recombinant PD-1 protein for 72 h. (**A**) MCF-7:Jurkat co-culture control; (**B**) MCF-7:Jurkat co-culture treated with 10 µg/mL PHA-M and 1 µg/mL human recombinant PD-1; (**C**) MCF-7:THP-1 co-culture control; (**D**) MCF-7:THP-1 co-culture treated with 1 µg/mL human recombinant PD-1; (**E**) MDA-MB-231:Jurkat co-culture control; (**F**) MDA-MB-231:Jurkat co-culture treated with 10 µg/mL PHA-M and 1 µg/mL human recombinant PD-1; (**G**) MDA-MB-231:THP-1 co-culture control; (**H**) MDA-MB-231:THP-1 co-culture treated with 1 µg/mL human recombinant PD-1. All experiments were repeated at least three times.

In our study, a co-culture model was established using hormone receptor-negative MDA-MB-231 breast cancer cells (ER^−^/PR^−^/HER2^−^) and estrogen receptor-positive MCF-7 breast cancer cells (ER^+^), along with THP-1 acute monocytic leukemia cells and/or Jurkat acute T lymphocyte leukemia cells. In this in vitro model, breast cancer and immune cells were plated at a 1:5 ratio to allow for direct cell–cell interaction. Furthermore, the PD-1 expression at basal levels was checked in the Jurkat and THP-1 cells to establish the co-culture model. Then, PD-1 expression was initially induced in the Jurkat cells using phytohemagglutinin-M (PHA-M) (10 µg/mL) (Supplementary Figures S1 and S2 and Table S1) and recombinant human PD-1 protein (1 µg/mL), and in the THP-1 cells using only recombinant human PD-1 protein (1 µg/mL) for 72 h [28]. Increased PD-1 expression was confirmed via flow cytometry (*p* < 0.001). The same concentration and incubation period were subsequently applied in all co-culture experiments (Figure 1).

### 2.2. Dox and PTX Treatments in the Breast Cancer Co-Culture Models

**Figure 2:**
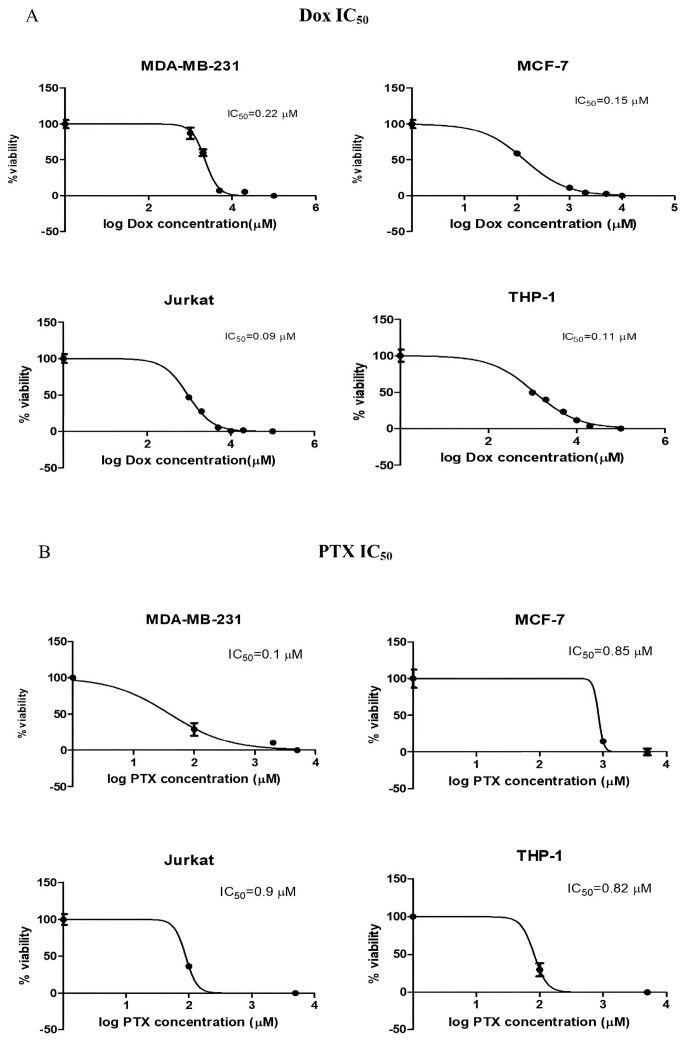
Dose–response curves and calculated IC_50_ values for (**A**) Dox and (**B**) PTX treatments in the MDA-MB-231, MCF-7, Jurkat, and THP-1 cell lines.

**Figure 3:**
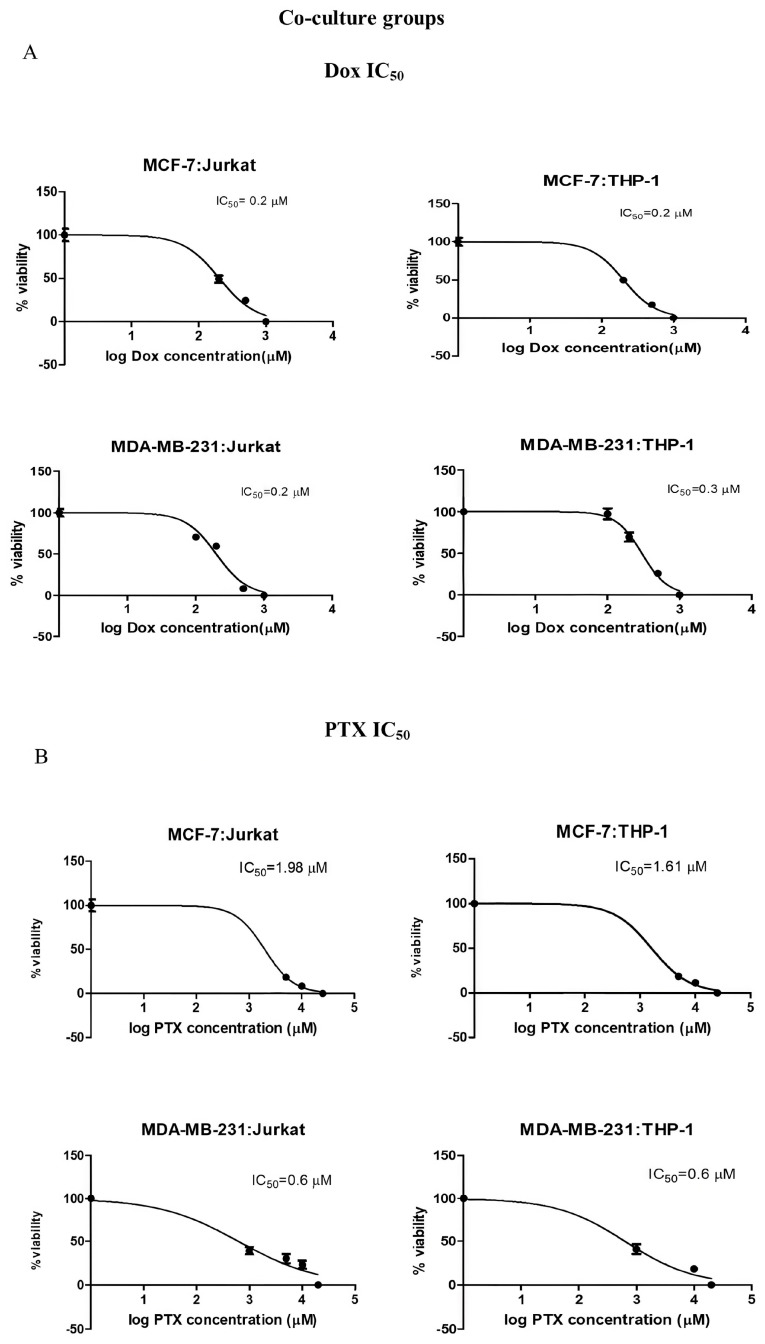
Dose–response curves and calculated IC_50_ values for (**A**) Dox and (**B**) PTX treatments in the co-culture groups (MCF-7:Jurkat, MCF-7:THP-1, MDA-MB-231:Jurkat, and MDA-MB-231:THP-1).

Dox was applied in the concentration range of 0.01–1 µM, while PTX was applied in the concentration range of 0.1–25 µM to all groups. A significant increase in IC_50_ values following PTX treatment was observed in the co-culture groups of both the MCF-7 and MDA-MB-231 cell lines (*p* < 0.001). Conversely, no significant change was observed in the Dox-treated groups (Figure 2 and Figure 3 and Table 1). According to these findings, 0.2 µM of Dox and 1 µM of PTX were determined as the concentrations to be used in the combination studies.

### 2.3. BMS-1166 and Its Effect on Breast Cancer Cell Viability

**Figure 4:**
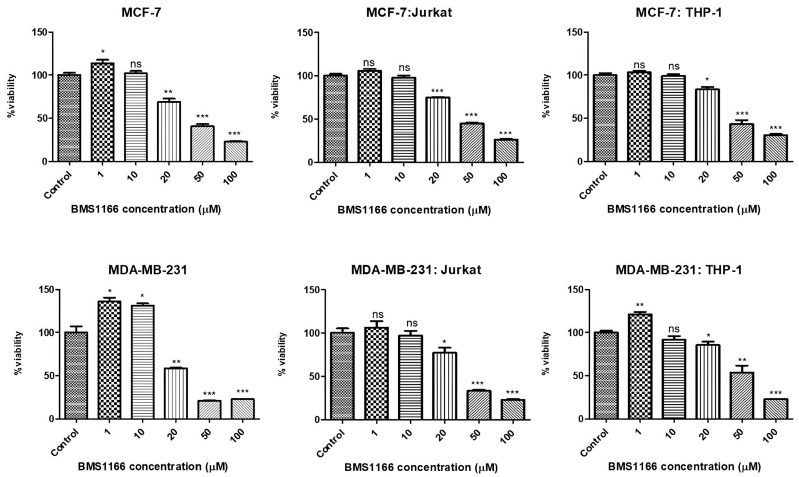
Effects of BMS-1166 on cell viability in the breast cancer cells and co-culture groups following 72 h treatment at concentrations ranging from 1 to 100 µM, as assessed by an MTT assay. *p* < 0.05 (*), *p* < 0.01 (**), *p* < 0.001 (***), and ns = non-significant.

Based on the viability results, the treatment dose of BMS-1166 was determined as 20 µM for 72 h (Figure 4 and Table 2). Representative cell viability images from BMS1166-treated breast cancer cells are included in Supplementary Figure S3.

### 2.4. Human PD-L1 Inhibitor IV and Its Effect on Breast Cancer Cell Viability

**Figure 5:**
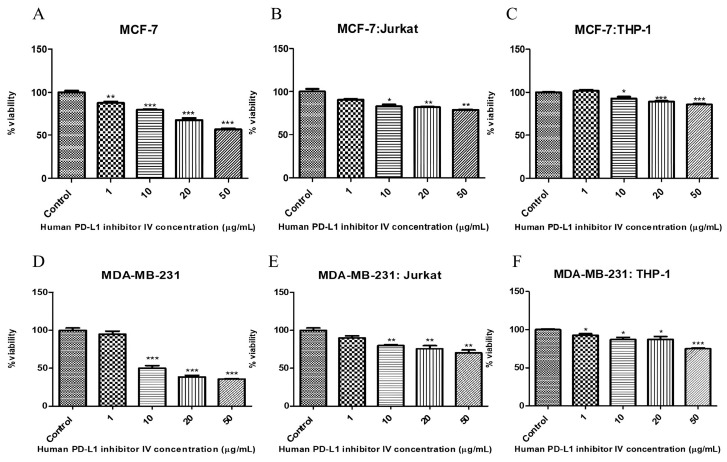
Effects of Human PD-L1 Inhibitor IV on cell viability in the breast cancer cells and co-culture groups following 72 h treatment at concentrations ranging from 1 to 50 µg/mL, as assessed by an MTT assay. (**A**) MCF-7; (**B**) MCF-7:Jurkat co-culture; (**C**) MCF-7:THP-1 co-culture; (**D**) MDA-MB-231; (**E**) MDA-MB-231:Jurkat co-culture; (**F**) MDA-MB-231:THP-1 co-culture. *p* < 0.05 (*), *p* < 0.01 (**), and *p* < 0.001 (***).

Considering the viability results of the MDA-MB-231 cells, a concentration of 10 µg/mL was selected as the appropriate dose for all cell groups (Figure 5 and Table 2). Representative cell viability images from Human PD-L1 Inhibitor IV-treated breast cancer cells are included in Supplementary Figure S4.

### 2.5. Cell Viability Analyses of the Combined Use of PD-1/PD-L1 Inhibitors and Chemotherapeutic Agents

**Figure 6:**
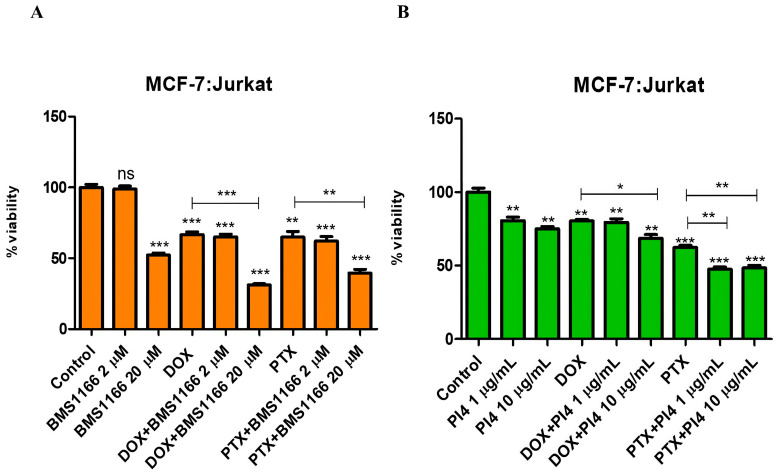
Cell viability results for the MCF-7:Jurkat co-culture group treated with various combinations of PD-1/PD-L1 inhibitors (BMS-1166 (2 and 20 µM) (**A**) and Human PD-L1 Inhibitor IV (PI4) (1 and 10 µg/mL) (**B**)) and chemotherapeutic agents (Dox (0.2 µM) and PTX (1 µM)), indicating the effects of combined immunotherapy and chemotherapy. Each experimental condition was performed in triplicate. *p* < 0.05 (*), *p* < 0.01 (**), and *p* < 0.001 (***); ns = non-significant.

**Figure 7:**
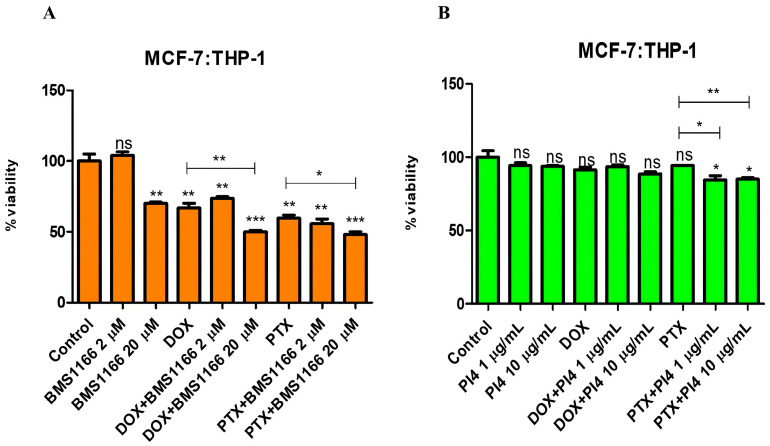
Cell viability results for the MCF-7:THP-1 co-culture group treated with various combinations of PD-1/PD-L1 inhibitors (BMS-1166 (2 and 20 µM) (**A**) and Human PD-L1 Inhibitor IV (PI4) (1 and 10 µg/mL) (**B**)) and chemotherapeutic agents (Dox (0.2 µM) and PTX (1 µM)), indicating the effects of combined immunotherapy and chemotherapy. Each experimental condition was performed in triplicate. *p* < 0.05 (*), *p* < 0.01 (**), and *p* < 0.001 (***); ns = non-significant.

**Figure 8:**
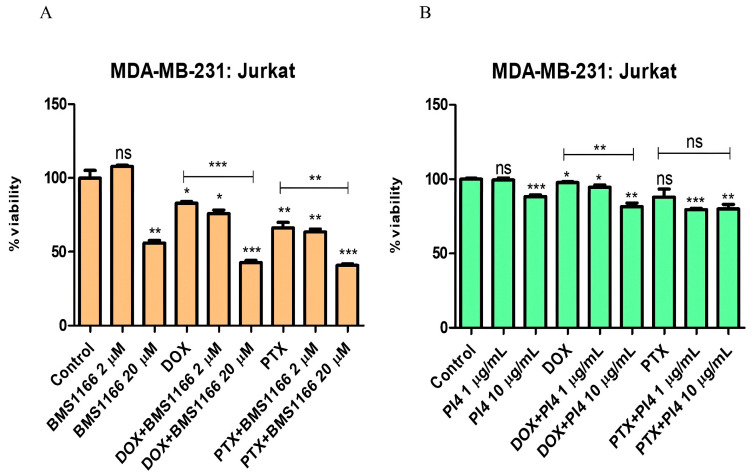
Cell viability results for the MDA-MB-231:Jurkat co-culture group treated with various combinations of PD-1/PD-L1 inhibitors (BMS-1166 (2 and 20 µM) (**A**) and Human PD-L1 Inhibitor IV (PI4) (1 and 10 µg/mL))(**B**)) and chemotherapeutic agents (Dox (0.2 µM) and PTX (1 µM)), indicating the effects of combined immunotherapy and chemotherapy. Each experimental condition was performed in triplicate. *p* < 0.05 (*), *p* < 0.01 (**), and *p* < 0.001 (***); ns = non-significant.

**Figure 9:**
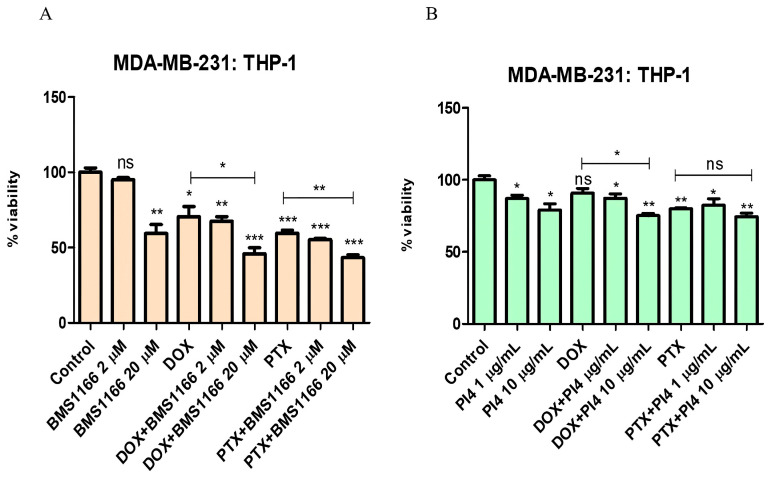
Cell viability results for the MDA-MB-231:THP-1 co-culture group treated with various combinations of PD-1/PD-L1 inhibitors (BMS-1166 (2 and 20 µM) (**A**) and Human PD-L1 Inhibitor IV (PI4) (1 and 10 µg/mL) (**B**)) and chemotherapeutic agents (Dox (0.2 µM) and PTX (1 µM)), indicating the effects of combined immunotherapy and chemotherapy. Each experimental condition was performed in triplicate. *p* < 0.05 (*), *p* < 0.01 (**), and *p* < 0.001 (***); ns = non-significant.

In the co-culture model established with Jurkat cells pre-stimulated for 72 h using 10 µg/mL of PHA-M, 1 µg/mL of recombinant human PD-1 protein, and breast cancer cells, PD-1/PD-L1 interaction was targeted using BMS-1166 at 2 µM and 20 µM and Human PD-L1 Inhibitor IV at 1 µg/mL and 10 µg/mL. Cell viability changes were assessed under combinational treatment conditions involving Dox (0.2 µM) and PTX (1 µM), together with low and high doses of the inhibitors (Figure 6, Figure 7, Figure 8 and Figure 9).

### 2.6. Drug Resistance and PD-1/PD-L1 Connection in Breast Cancer

Drug resistance proteins were analyzed in all co-culture groups, together with PD-1/PD-L1 interaction. The effects of inhibitor and chemotherapy combinations were compared to both the control group and the individual chemotherapy treatments.

**Figure 10:**
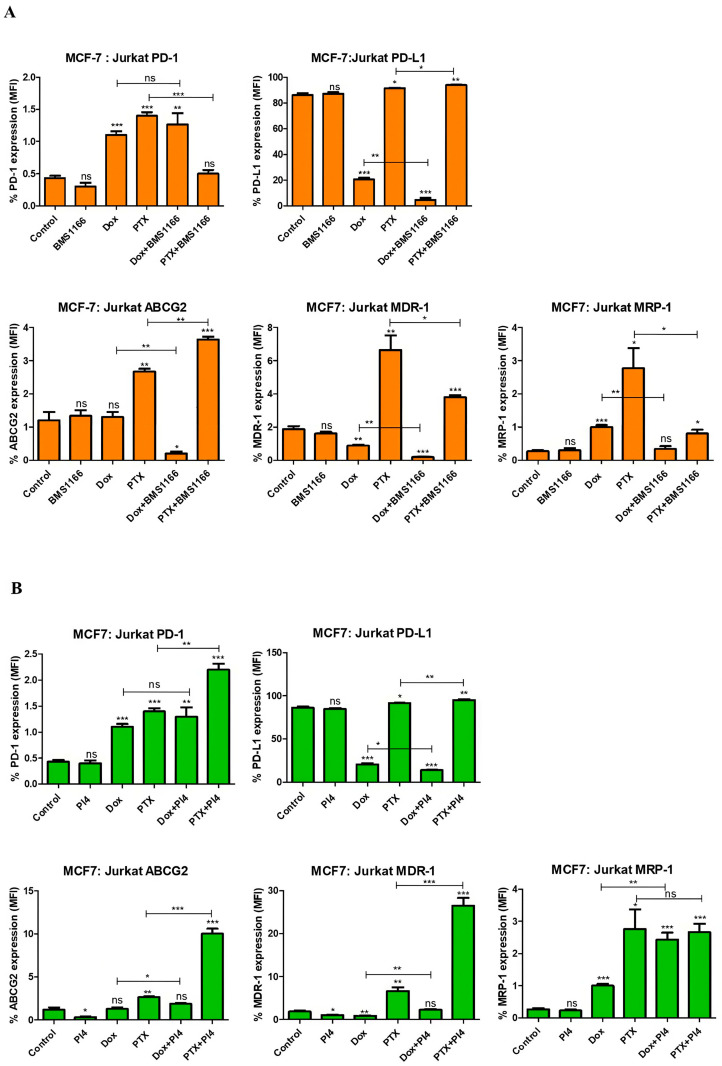
Flow cytometric analysis of PD-1, PD-L1, ABCG2, MDR-1, and MRP-1 expression levels following treatment with BMS-1166 (**A**) and Human PD-L1 Inhibitor IV (PI4) (**B**), applied either alone or in combination with chemotherapeutic agents in the MCF-7:Jurkat co-culture. Each experimental condition was performed in triplicate. *p* < 0.05 (*), *p* < 0.01 (**), and *p* < 0.001 (***); ns = non-significant.

In the MCF-7:Jurkat co-culture, BMS-1166 treatment alone did not significantly alter the expression of PD-1 (*p* = 0.12), PD-L1 (*p* = 0.61), or drug resistance proteins compared to the control. Dox and PTX demonstrated distinct immunomodulatory and drug resistance-related effects, with PTX markedly increasing the expression of all evaluated markers. Notably, the combination of chemotherapeutics with BMS-1166 modulated these effects in a drug-dependent manner, suggesting that BMS-1166 may enhance or counteract chemotherapy-induced changes in PD-1/PD-L1 and resistance protein expression within the tumor immune microenvironment (Figure 10A).

Human PD-L1 Inhibitor IV alone demonstrated limited impact on marker expression, whereas its combination with chemotherapeutic agents resulted in more pronounced alterations. Notably, co-treatment with PTX led to significant increases in PD-1/PD-L1 and drug resistance protein levels, suggesting that the inhibitor’s effects may be context-dependent and modulated by chemotherapy. This result suggests that PD-L1 inhibitors may contribute to alterations in resistance-associated protein expression when used in combination with chemotherapeutic agents (Figure 10B).

**Figure 11:**
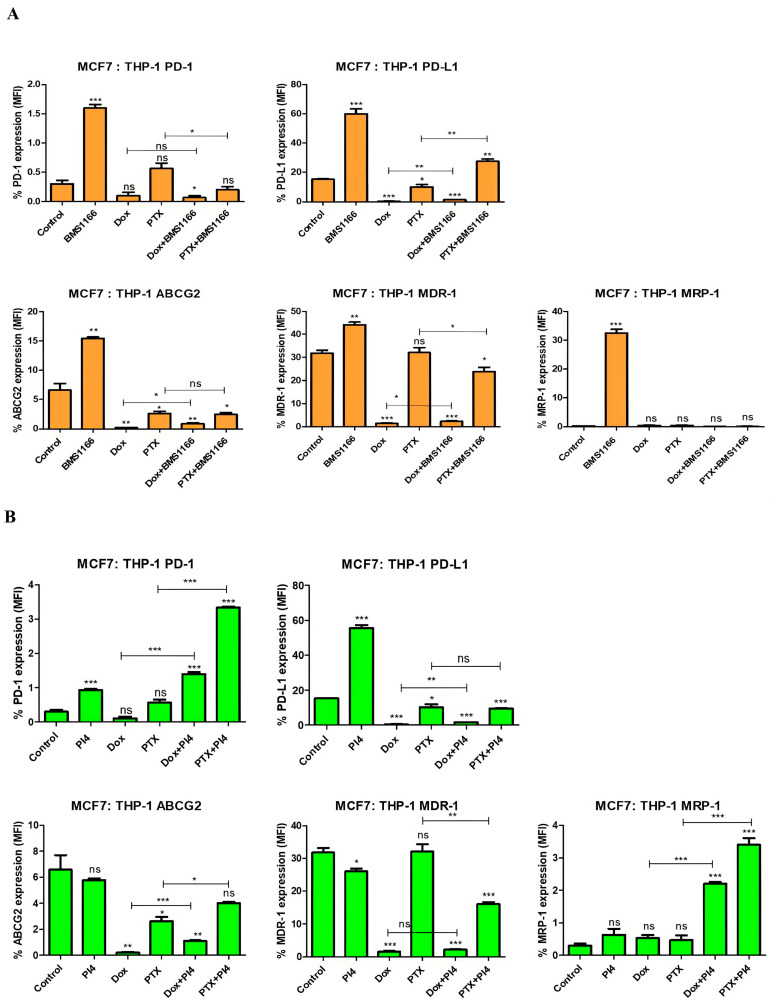
Flow cytometric analysis of PD-1, PD-L1, ABCG2, MDR-1, and MRP-1 expression levels following treatment with BMS-1166 (**A**) and Human PD-L1 Inhibitor IV (PI4) (**B**), applied either alone or in combination with chemotherapeutic agents in the MCF-7:THP-1 co-culture. Each experimental condition was performed in triplicate. *p* < 0.05 (*), *p* < 0.01 (**), and *p* < 0.001 (***); ns = non-significant.

In the MCF-7:THP-1 co-culture model, BMS-1166 alone significantly increased the expression of PD-1 (*p* < 0.001), PD-L1 (*p* < 0.001), and drug resistance proteins (ABCG2 (*p* = 0.002); MDR-1 (*p* = 0.002); MRP-1 (*p* < 0.001)) compared to the control, whereas both Dox and PTX alone led to a marked reduction in several key markers, particularly PD-L1 (*p* < 0.001; *p* = 0.04) and ABCG2 (*p* = 0.005; *p* = 0.03), respectively. When combined with BMS-1166, the chemotherapeutics produced differential effects, with BMS-1166 appearing to partially reverse or modulate the marker downregulation induced by Dox, while enhancing certain PTX-associated changes (Figure 11A). These results suggest a complex interaction between BMS-1166 and chemotherapeutic agents in regulating immune and resistance-related protein expression within the tumor–macrophage microenvironment.

Human PD-L1 Inhibitor IV alone led to increased expression of PD-1 (*p* < 0.001), PD-L1 (*p* < 0.001), and decreased expression of MDR-1 (*p* = 0.02) (Figure 11B), while showing no significant effect on ABCG2 (*p* = 0.51) or MRP-1 (*p* = 0.16). Chemotherapeutic agents alone, particularly Dox and PTX, caused a general downregulation in PD-L1 (*p* < 0.001; *p* = 0.04) and ABCG2 (*p* = 0.005; *p* = 0.03), respectively. Interestingly, combination treatments with the PD-L1 inhibitor showed variable effects depending on the drug, in some cases reversing, enhancing, or dampening chemotherapy-induced changes (Figure 11B). Thus, these findings suggest a dynamic interplay between immune checkpoint inhibition and chemotherapy in modulating both immunological and drug resistance markers in the tumor–macrophage interface.

**Figure 12:**
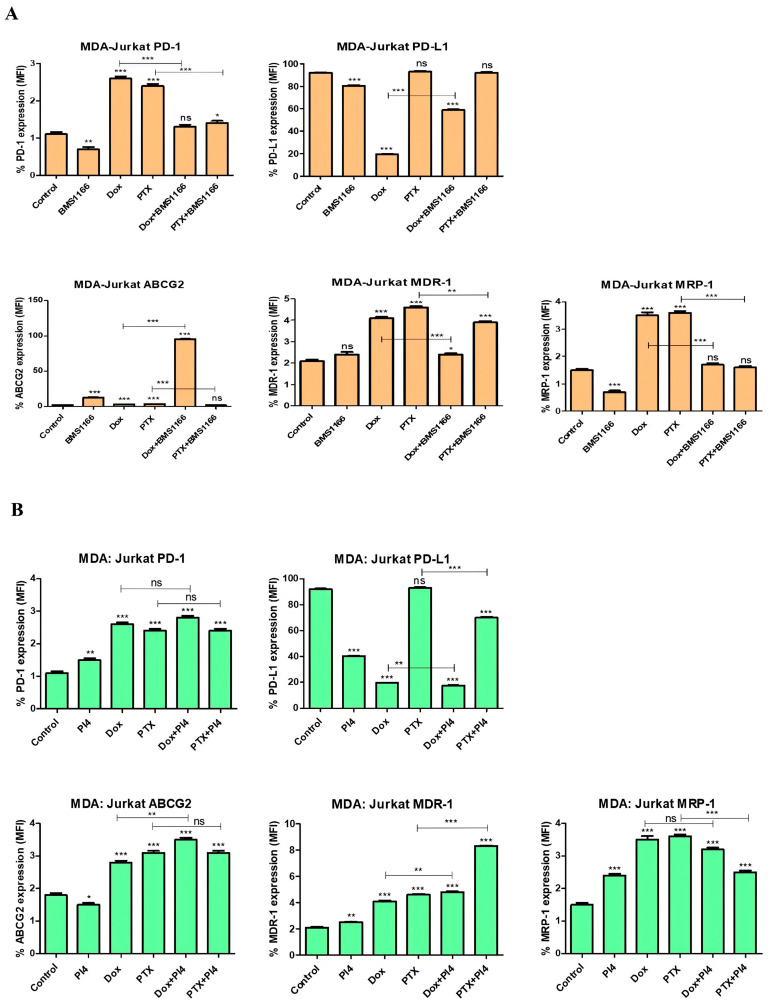
Flow cytometric analysis of PD-1, PD-L1, ABCG2, MDR-1, and MRP-1 expression levels following treatment with BMS-1166 (**A**) and Human PD-L1 Inhibitor IV (PI4) (**B**), applied either alone or in combination with chemotherapeutic agents in the MDA-MB-231:Jurkat co-culture. Each experimental condition was performed in triplicate. *p* < 0.05 (*), *p* < 0.01 (**), and *p* < 0.001 (***); ns = non-significant.

In the MDA-MB-231:Jurkat co-culture model, BMS-1166 alone resulted in a significant downregulation of PD-1 (*p* = 0.008), PD-L1 (*p* < 0.001), and MRP-1 (*p* < 0.001), while inducing an increase in ABCG2 expression (*p* < 0.001). Dox and PTX each led to distinct upregulation patterns in drug resistance markers and PD-1, with Dox reducing PD-L1 levels. The combination treatments revealed a modulatory effect of BMS-1166 on chemotherapy-induced changes, suggesting that its impact is highly context-dependent and may help fine-tune immune and resistance responses in triple-negative breast cancer treatment (Figure 12A).

Combined treatment with Human PD-L1 Inhibitor IV and chemotherapeutic agents showed distinct modulation of drug resistance-related proteins. Notably, PD-L1 expression was consistently decreased when the inhibitor was used (*p* < 0.001), while ABCG2 (*p* = 0.02), MDR-1 (*p* = 0.008), and MRP-1 (*p* < 0.001) levels were elevated, including in the combination groups. Our results indicate that PD-L1 blockade may influence the expression of key resistance markers in the breast cancer microenvironment (Figure 12B).

**Figure 13:**
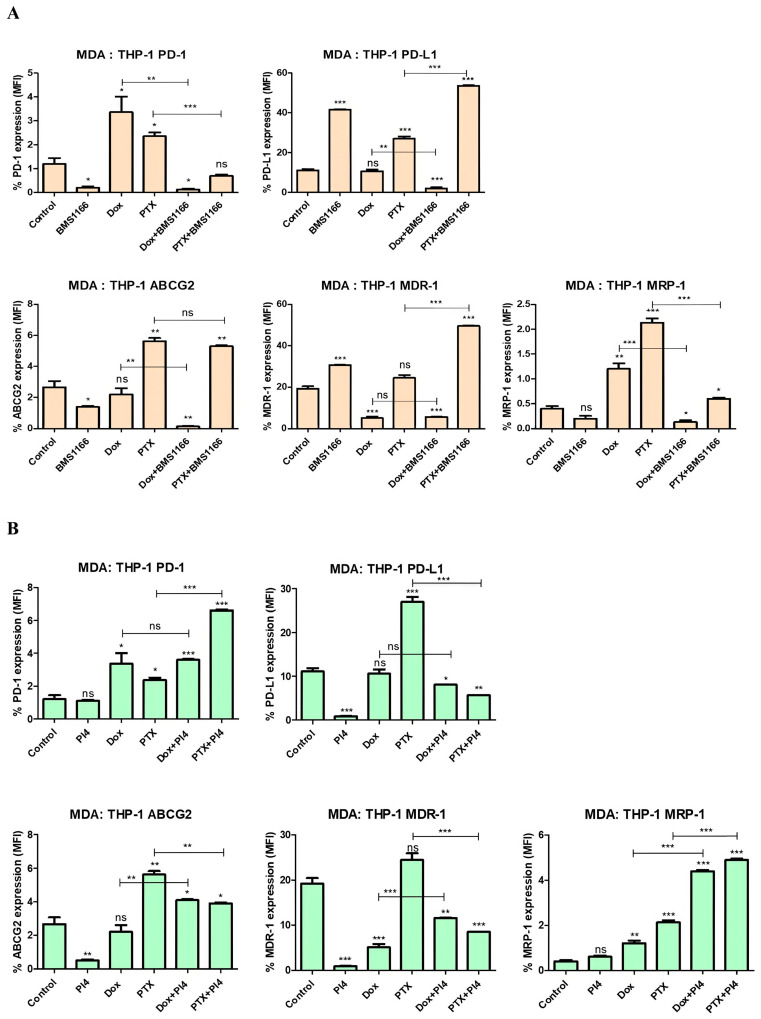
Flow cytometric analysis of PD-1, PD-L1, ABCG2, MDR-1, and MRP-1 expression levels following treatment with BMS-1166 (**A**) and Human PD-L1 Inhibitor IV (PI4) (**B**), applied either alone or in combination with chemotherapeutic agents in the MDA-MB-231:THP-1 co-culture. Each experimental condition was performed in triplicate. *p* < 0.05 (*), *p* < 0.01 (**), and *p* < 0.001 (***); ns = non-significant.

In the MDA-MB-231:THP-1 co-culture model, the data demonstrate that BMS-1166 exerted a bidirectional modulatory effect on both immune checkpoint and drug resistance markers. While BMS-1166 alone led to the downregulation of PD-1 (*p* = 0.02) and ABCG2 (*p* = 0.04), it upregulated PD-L1 (*p* < 0.001) and MDR-1 (*p* < 0.001) expression. In contrast, Dox and PTX induced different expression patterns, and when combined with BMS-1166, distinct drug-specific interactions were observed, such as attenuation of Dox-induced effects and enhancement of PTX-related changes in certain markers. These findings underscore the context-specific behavior of immunotherapeutic modulation within the tumor microenvironment (Figure 13A).

Based on the results, Human PD-L1 Inhibitor IV alone led to a significant reduction in PD-L1 (*p* < 0.001), ABCG2 (*p* = 0.006), and MDR-1 (*p* < 0.001) expression, without affecting PD-1 (*p* = 0.71) or MRP-1 (*p* = 0.07) levels. Dox and PTX induced distinct changes, with Dox increasing PD-1 (*p* = 0.04) and MRP-1 (*p* = 0.003), while PTX broadly upregulated most markers except MDR-1 (*p* = 0.05). Combination treatments revealed complex interactions, where PD-L1 inhibition modulated chemotherapy-induced expression patterns, either enhancing or suppressing specific drug-affected targets depending on the agent used (Figure 13B).

## 3. Discussion

Immunotherapy approaches are becoming increasingly common alongside conventional treatments in the management of breast cancer [29]. A variety of in vitro cell culture approaches have been utilized to elucidate the complex mechanisms underlying cancer biology. In our study, we investigated the relationship between PD-1 and PD-L1 molecules and drug resistance in the context of chemotherapy- and immunotherapy-focused treatments, using an in vitro breast cancer model developed with monocyte and lymphocyte cells to explore the immune basis of breast cancer. The in vitro co-culture model was designed with an immune cell-to-cancer cell ratio of 5:1, representing a condition in which the immune system is relatively inadequate [30,31].

In the model we established, to provide preclinical data for both tumor microenvironment and immunotherapy studies related to the interaction between breast cancer and immune cells, T cell activation was initially achieved using PHA-M (10 μg/mL) and recombinant human PD-1 protein (1 µg/mL) for 72 h, thus increasing PD-1 expression levels (Supplementary Figures S1–S3 and Table S1) (*p* < 0.001). A previous study, consistent with our findings, demonstrated that co-culturing HepG2 liver cancer cells with Jurkat cells and stimulating T cells using 2 µg/mL of PHA for 48 h resulted in optimal Jurkat cell activation [32].

In the present study, the PD-1/PD-L1 interaction was inhibited using two different inhibitors. The treatments of these inhibitors with the chemotherapeutic agents Dox and PTX were evaluated.

Initially, the IC_50_ values of Dox and PTX were evaluated for 72 h in all cell lines and co-culture models, and the variations in IC_50_ values within the co-culture groups are summarized in Table 1. In the co-culture groups established with MDA-MB-231 cells, no significant change in IC_50_ values was observed following Dox treatment, whereas a notable increase in IC_50_ values was detected with PTX treatment (from 0.1 to 0.6 µM; *p* < 0.001). In comparison, for the MCF-7 cell co-cultures, Dox treatment did not lead to a significant change in IC_50_ values, while PTX treatment led to an approximate twofold increase in IC_50_ values in the MCF-7:Jurkat and MCF-7:THP-1 co-cultures (Figure 2 and Table 1). Considering the increased IC_50_ values observed in MCF-7 cells and their respective immune cell co-cultures, PTX appears to have the potential for inducing resistance. A similar pattern was also evident in the MDA-MB-231 co-culture groups (Figure 2 and Figure 3 and Table 1) (*p* < 0.001). Furthermore, the cytotoxic effects of the BMS-1166 and PI4 inhibitors were assessed in the breast cancer co-culture models. When considered collectively, the average IC_50_ dose for BMS-1166 across all cell lines was determined to be approximately 20 µM following 72 h of treatment. The IC_50_ value of PI4 was identified exclusively for MDA-MB-231 cells as 10 µg/mL and was applied in further experiments (Table 2).

In our study, Dox treatment significantly reduced PD-L1 expression and induced its inhibition across all co-culture groups, with the exception of the MDA-MB-231:THP-1 co-culture (Figure 10, Figure 11, Figure 12 and Figure 13) (*p* = 0.68). Similarly, several studies have demonstrated that Dox treatment significantly decreases PD-L1 expression in MDA-MB-231 cells, which aligns with our findings [33,34]. This parallel supports the notion that Dox may directly modulate immune checkpoint expression, contributing to its immunogenic effects. A study on breast cancer demonstrated that silencing PD-L1 enhances the apoptotic effects of Dox. The same study proposed that PD-1/PD-L1 inhibition may improve Dox efficacy by suppressing MDR1/P-glycoprotein (P-gp) expression in breast cancer cells [23]. PTX, on the contrary, exhibited varying effects on PD-L1 expression across different co-culture groups.

In another study, flow cytometric analysis revealed increased PD-L1 protein expression in MDA-MB-468, MDA-MB-435, and MCF-7 breast cancer cell lines following treatment with PTX, etoposide, and 5-fluorouracil [35]. In our study, PTX-induced upregulation of PD-L1 was detected in the MCF-7:Jurkat co-culture model (Figure 10) (*p* = 0.02).

Additionally, the combination of Dox with BMS-1166 and PI4 resulted in a significant reduction in PD-L1 expression (Figure 12) (*p* < 0.001). The BMS-1166 and PI4 inhibitors primarily target the PD-1/PD-L1 signaling pathway. Accordingly, a notable decrease in PD-L1 expression induced by both inhibitors was specifically observed in the MDA-MB-231:Jurkat co-culture group (Figure 12) (*p* < 0.001). This observation is consistent with clinical patient data. MDA-MB-231 is a TNBC cell line with an aggressive, metastatic molecular profile, while Jurkat cells exhibit the characteristics of CD4^+^ T lymphocytes. Notably, immunotherapy is typically administered to patients with advanced-stage TNBC, and the significance of TIL (tumor-infiltrating lymphocyte) levels and PD-L1 positivity as criteria for immunotherapy eligibility is frequently emphasized [36]. Therefore, the MDA-MB-231:Jurkat co-culture model may serve as a representative system for understanding the clinical course.

Multidrug resistance to a broad spectrum of anticancer drugs is widely recognized as a key factor underlying treatment failure and remains a significant barrier to the effective management of many cancer types. One of the main mechanisms contributing to MDR is the increased efflux of drugs driven by adenosine triphosphate (ATP). This process typically involves cellular transport proteins that actively pump drugs out of the cell. Among these, ATP-binding cassette (ABC) transporters represent a major group responsible for drug efflux in an energy-dependent manner. Well-characterized members of this transporter family include P-glycoprotein (P-gp, also known as MDR-1 or ABCB-1), MRP-1 or ABCC-1, and BCRP or ABCG2, all of which are commonly overexpressed in drug-resistant cancer cells. It is known that PTX and Dox are substrates of MDR-1 [37,38]. As a peptide-based inhibitor, PI4 may also have the potential to be a substrate of MDR-1, which is a member of the ABC transporter family [39]. In our study, PI4 treatment resulted in a decrease in MDR-1 expression in all co-culture groups except for the MDA-MB-231:Jurkat co-culture (Figure 10, Figure 11, Figure 12 and Figure 13). The BMS-1166 inhibitor is known to contain both hydrophobic and hydrophilic side groups. Moreover, due to its small molecular structure, it has the potential to interact with various drug resistance proteins [40]. To the best of our knowledge, no studies have been published to date that investigate the interaction between BMS-1166 or PI4 and drug resistance. Only one study has examined low-dose BMS-1166 treatment under conditions of induced resistance in colorectal cancer cells, although this study did not evaluate the expression of proteins associated with drug resistance [41].

In our study, treatment with BMS-1166 alone led to an increase in MDR-1 expression in the MCF-7:THP-1 (*p* = 0.002) and MDA-MB-231:THP-1 (*p* < 0.001) co-culture groups, whereas no significant change was observed in the MCF-7:Jurkat (*p* = 0.29) or MDA-MB-231:Jurkat (*p* = 0.08) co-culture groups compared to the control (Figure 10, Figure 11, Figure 12 and Figure 13). The present data highlight that the observed increase in MDR-1 expression may be mediated by THP-1 cells, indicating that the monocyte population could contribute to the development of drug resistance [42].

In the MDA-MB-231:Jurkat co-culture, ABCG2 expression increased (*p* < 0.001), MDR-1 expression remained unchanged (*p* = 0.08), and MRP-1 expression decreased (*p* < 0.001). Conversely, in the MDA-MB-231:THP-1 co-culture, ABCG2 expression decreased (*p* = 0.03), MDR-1 expression increased (*p* < 0.001), and no change was detected in MRP-1 expression (*p* = 0.07). These findings suggest that BMS-1166 does not produce consistent results across MDA-MB-231 co-culture groups, and that additional resistance-related parameters may need to be investigated in this regard (Figure 12 and Figure 13). Both inhibitors were found to reduce PD-L1 expression; however, BMS-1166 increased the expression of the drug resistance protein ABCG2 while decreasing MRP-1, whereas PI4 upregulated the expression of ABCG2, MDR-1, and MRP-1. Overall, it can be suggested that while both inhibitors decrease PD-L1 expression, they may simultaneously contribute to increased drug resistance. In the MDA-MB-231:Jurkat co-culture group, the combination of BMS-1166 and PTX reduced PTX-induced drug resistance proteins (Figure 12). Strikingly, in the MDA-MB-231:Jurkat co-culture, the Dox + BMS-1166 (*p* < 0.001) and PTX + BMS-1166 (*p* = 0.001) combinations led to a marked reduction in MDR-1 expression, whereas the Dox + PI4 (*p* = 0.001) and PTX + PI4 (*p* < 0.001) combinations caused a clear increase when compared separately with their respective monotherapies (Dox or PTX alone). Despite both inhibitors effectively lowering PD-L1 expression, they triggered opposing outcomes on MDR-1 expression, strongly indicating that this divergence may result from mechanisms unrelated to PD-L1 inhibition.

A previous study demonstrated a positive correlation between PD-L1 and MDR-1 expression in breast cancer tissues, suggesting that this relationship may contribute to chemotherapy resistance [43]. In our study, similar to the tissue findings, PTX and Dox treatments alone showed parallel PD-L1 and MDR-1 expression patterns in all co-culture groups, with the exception of the MDA-MB-231:Jurkat group (Figure 10, Figure 11, Figure 12 and Figure 13).

Overall, our findings suggest that the inhibition of the PD-1/PD-L1 axis may be effective in induction chemotherapy without the development of drug resistance in cancer, as it provides effective immune checkpoint blockade and thus may be a new approach to increase the first-line efficacy of chemotherapy. This study uniquely evaluated both PD-1/PD-L1 blockade and drug resistance within an in vitro breast cancer model, combinations of checkpoint inhibitors, and chemotherapeutic agents. These results highlight the potential role of immune cells in modulating drug resistance [44] and immune checkpoint expression, underscoring the importance of combinatorial strategies in breast cancer therapy.

## 4. Materials and Methods

### 4.1. Cell Culture

MCF-7 (breast adenocarcinoma), MDA-MB-231 (metastatic breast adenocarcinoma), Jurkat (acute T cell leukemia), and THP-1 (acute monocytic leukemia) cell lines were obtained from the American Type Culture Collection (ATCC (Manassas, VA, USA)). The MCF-7 and MDA-MB-231 cells were maintained in Dulbecco’s modified Eagle medium (DMEM) low-glucose (Sigma-Aldrich (St. Louis, MO, USA); Cat#D2902) medium. The Jurkat and THP-1 cell lines were maintained in Roswell Park Memorial Institute (RPMI) (Sigma-Aldrich; Cat#R6504) medium. All media were supplemented with 10% fetal bovine serum (FBS) (Hyclone; Cytiva (Logan, UT, USA); Cat#SV30160.03) and 1% penicillin/streptomycin (Gibco (Thermo Fisher Scientific (Waltham, MA, USA); Cat#15140122). Cells were grown at 37 °C under 5% CO_2_ atmosphere. To stimulate immune cells and mimic the presence of PD-1, phytohemagglutinin-M (PHA-M) (Biosera (Nuaille, France); Cat#LM-T1740/5) and human recombinant PD-1 (SinoBiological (Beijing, China); Cat#10377-H02H) were used. As anticancer drugs, Dox (Glentham Life Sciences (Corsham, UK); Cat#GA4969) and PTX (Glentham Life Sciences; Cat#GA4564) were purchased. The PD-L1 inhibitor BMS-1166 (Selleckchem (Houston, TX, USA); Cat#S8859) and the PD-1 inhibitor Human PD-L1 Inhibitor IV (Anaspec Inc. (Fremont, CA, USA); Cat#AS-65584) were used in this study. The concentrations of the drugs and inhibitors used in this study were as follows: Dox was prepared at 10 mM in DMSO; PTX at 1 mM in DMSO; BMS1166 at 5 mM in DMSO; and Human PD-L1 Inhibitor IV was prepared as a stock solution at 1 mg/mL in sterile water. Working concentrations were obtained by serially diluting these stock solutions in the culture medium.

### 4.2. In Vitro Co-Culture Model

Co-culture models were established comprising hormone receptor-negative MDA-MB-231 (ER^−^/PR^−^/HER2^−^) and estrogen receptor-positive MCF-7 (ER^+^) breast cancer cell lines, in combination with THP-1 acute monocytic leukemia cells or Jurkat acute T lymphoblastic leukemia cells as immune cells. The Jurkat cell line was stimulated with PHA-M and human recombinant PD-1 protein. Co-cultures with THP-1 cells were supplemented with human recombinant PD-1 protein to induce PD-1 expression. Activated and PD-1-expressing Jurkat and THP-1 cells were added to the breast cancer cells at an effector-to-target (E/T) ratio of 5:1 and co-cultured for 72 h.

### 4.3. Cell Viability Assay

To prepare the MTT stock solution, the reagent was dissolved in PBS at a concentration of 5 mg/mL. Cells were seeded on 96-well plates (MCF-7 and MDA-MB-231 breast cancer cells at 2000 cells/well, Jurkat and THP-1 cells at 10,000 cells/well) for co-culture (1:5 cell ratio). After administering the test agents for 72 h, the assay was initiated by adding 10 μL of the MTT stock solution to each well of the 96-well plate, resulting in a final working concentration of 0.5 mg/mL (1:10 dilution). The plates were then incubated at 37 °C for 3 to 3.5 h. Following incubation, the culture medium was carefully removed, and the resulting formazan crystals were solubilized with DMSO. After achieving a homogeneous solution, absorbance was measured at 570 nm using the Allsheng FlexA-200 microplate reader (Hangzhou Allsheng Instruments Co., Ltd., Hangzhou, China) [45]. All experiments were repeated at least three times. Using the MTT assay results, we calculated the cytotoxicity percentages across various concentrations, as well as the half-maximal inhibitory concentration (IC_50_) values for each tested drug and inhibitor following 72 h of treatment, utilizing GraphPad Prism 9 software.

### 4.4. Flow Cytometry

The PD-1, PD-L1, MDR-1, MRP-1, and ABCG2 drug resistance-related proteins were assessed using flow cytometry. The following antibodies were used in this study: PD-1 (PE-conjugated; BioLegend, San Diego, CA, USA; Cat# 329906), PD-L1 (PE-conjugated; BioLegend; Cat# 329706), MDR-1 (PE-conjugated; BioLegend; Cat# 348606), ABCG2 (PE-conjugated; BioLegend; Cat# 332008), and MRP-1 (Alexa Fluor 488-conjugated; BioLegend; Cat# 370306). The cells were processed under two different conditions: individually and in co-culture. Initially, breast cancer cells were cultured in 6-well plates and allowed to adhere for 24 h, after which immune cells were simultaneously added. Chemotherapeutic agents and inhibitors were then administered at their respective IC_50_ concentrations. The co-cultures were incubated for 72 h under standard conditions. At the end of incubation, the cells were detached by trypsinization and collected into centrifuge tubes. The cell suspension was centrifuged at 300× *g* for 10 min, after which the supernatant was discarded, and the cell pellet was washed twice with PBS. Subsequently, the cells were counted and resuspended in PBS. Then, 5 μL of antibody was added to 100 μL of the resuspended cells, which were vortexed to ensure proper mixing. The samples were incubated for 30 min at +4 °C in the dark. After incubation, 400 μL of PBS was added, and the tubes were centrifuged at 2000 rpm for 5 min. The supernatant was discarded, and the cells were resuspended in 500 μL of PBS in flow cytometry tubes. All experiments were repeated at least three times. All samples were analyzed by acquiring a minimum of 10,000 events per sample using the Navios EX flow cytometer (Beckman Coulter, Pasadena, CA, USA). Data were analyzed using Navios EX Software (version 2.0, Beckman Coulter).

### 4.5. Statistical Analysis

All statistical analyses were performed using GraphPad Prism version 9 software. The Kolmogorov–Smirnov test was used to assess the normality of data distribution. Unpaired Student’s *t*-test was applied for comparisons between two groups and to evaluate differences relative to the control group. All experiments were conducted with at least three independent replicates. A *p*-value of <0.05 was considered statistically significant. Statistical significance levels are indicated as follows: *p* < 0.05 (*), *p* < 0.01 (**), and *p* < 0.001 (***).

## 5. Conclusions

The results obtained from this study are significant because they demonstrate that chemotherapy drugs and PD-1/PD-L1-targeted inhibitors have different effects on the expression of PD-L1 and drug resistance-related proteins in breast cancer, particularly in tumors with varying immune cell populations.

The current research provides several novel insights into the dynamic interplay between immune checkpoint inhibition, chemotherapeutic agents, and drug resistance in breast cancer. In this study, using co-culture models with distinct immune cell types (Jurkat T cells and THP-1 monocytes), we demonstrated that the cellular immune context significantly influences PD-1/PD-L1 signaling and drug resistance protein expression. Notably, both BMS-1166 and Human PD-L1 Inhibitor IV not only modulated PD-1/PD-L1 expression but also altered the levels of ABCG2, MDR-1 and MRP-1, revealing a broader impact of immunotherapy on resistance mechanisms. Furthermore, the combination of immune checkpoint inhibitors with Dox or PTX exhibited drug-dependent modulatory effects, either enhancing or attenuating the expression of specific markers, highlighting the sensitive nature of these interactions. Using the TNBC cell line MDA-MB-231, known for its aggressive and treatment-resistant phenotype, enhances the translational relevance of our findings. Collectively, our results underscore the importance of immune microenvironment composition in shaping therapeutic responses and offer new perspectives on the integration of immunotherapy into drug-resistant cancer treatment strategies.

## Figures and Tables

**Table 1:** Comparison of the IC_50_ concentrations of Dox and PTX between the cell lines and co-culture groups.

Cells/Co-Culture Groups	DOX	PTX
**MCF-7**	0.15 µM	0.85 µM
**MDA-MB-231**	0.22 µM	0.1 µM
**Jurkat**	0.09 µM	0.9 µM
**THP-1**	0.11 µM	0.82 µM
**MCF-7:Jurkat**	0.2 µM	1.98 µM
**MCF-7:THP-1**	0.2 µM	1.61 µM
**MDA-MB-231:Jurkat**	0.2 µM	0.6 µM
**MDA-MB-231:THP-1**	0.3 µM	0.6 µM

The IC_50_ values for Dox and PTX after 72 h of treatment were determined using GraphPad Prism 9 software.

**Table 2:** IC_50_ values of BMS-1166 and Human PD-L1 Inhibitor IV treatment in the breast cancer cells and co-culture groups.

Cells/Co-Culture Groups	BMS-1166	PI4
**MCF-7**	21.7 µM	>50 µg/mL
**MCF-7:Jurkat**	28.7 µM	>50 µg/mL
**MCF-7:THP-1**	30 µM	>50 µg/mL
**MDA-MB-231**	17.6 µM	10 µg/mL
**MDA-MB-231:Jurkat**	24.7 µM	>50 µg/mL
**MDA-MB-231:THP-1**	24.5 µM	>50 µg/mL

The breast cancer cells and co-culture groups were treated with BMS-1166 and Human PD-L1 Inhibitor IV for 72 h to determine the half-maximal inhibitory concentration (IC_50_) values using GraphPad Prism software. The resulting data are presented in Figure 4 and Figure 5.

## Data Availability

Data is contained within the article or Supplementary Materials.

## Supplementary Materials

The following supporting information can be downloaded at: https://www.mdpi.com/article/10.3390/ijms26146876/s1.
