# Supplementary material for: PD-1/PD-L1 Inhibitors and Chemotherapy Synergy: Impact on Drug Resistance and PD-L1 Expression in Breast Cancer-Immune Cell Co-Cultures
Source: Int J Mol Sci. 2025 Jul 17;26(14):6876. doi: 10.3390/ijms26146876 (PMC12295165; doi:10.3390/ijms26146876)
Supplement: Supplementary file 1 [file ijms-26-06876-s001.zip › ijms-3713959-supplementary.pdf]

Supplementary material

Basal PD-1 Expression Levels in Jurkat and THP-1 Cell Lines

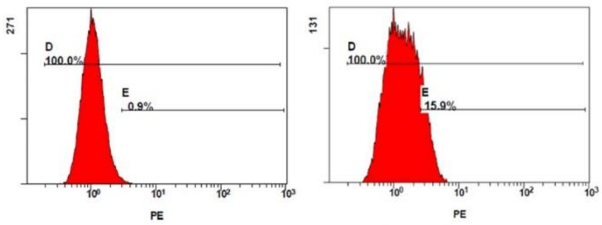

Jurkat-PD-1 THP-1-PD-1

Figure S1. Flow cytometry analysis of basal PD-1 expressions Jurkat and THP-1 cell line

PD-1 activation in Jurkat cells with PHA-M

PHA-M was incubated with Jurkat cells for 72 hours at concentrations ranging from 0.1 to 10 µg/mL. PD-1 expression levels at the determined appropriate doses were assessed by flow cytometry.

Table S1. Flow cytometry analysis of PD-1 expression changes in Jurkat cells treated with different concentrations of PHA-M compared to control

**Jurkat**		**PHA-M**	
	**1 µg/mL**	**5 µg/mL**	**10 µg/mL**
**PD-1 expression**	**% 0,1**	**%4,3**	**%9,6**

A B

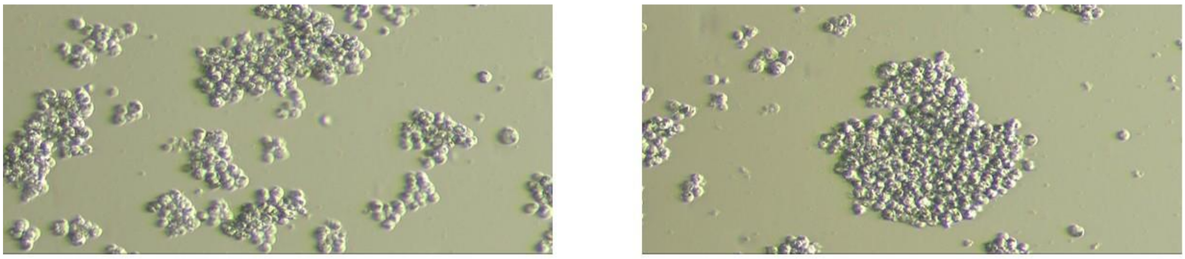

Jurkat 72 hour Control

Jurkat 72 hour PHA-M 10μg/mL

Figure S2. Representative image of Jurkat cell activation following 72-hour treatment with 10 μg/mL PHA-M.

A concentration of 10 μg/mL was identified as the optimal dose for PHA-M stimulation.

The addition of recombinant PD-1 protein into the co-culture system, including the THP-1 group, was deemed appropriate for the experimental design in order to enhance PD-1 expression in the cells.

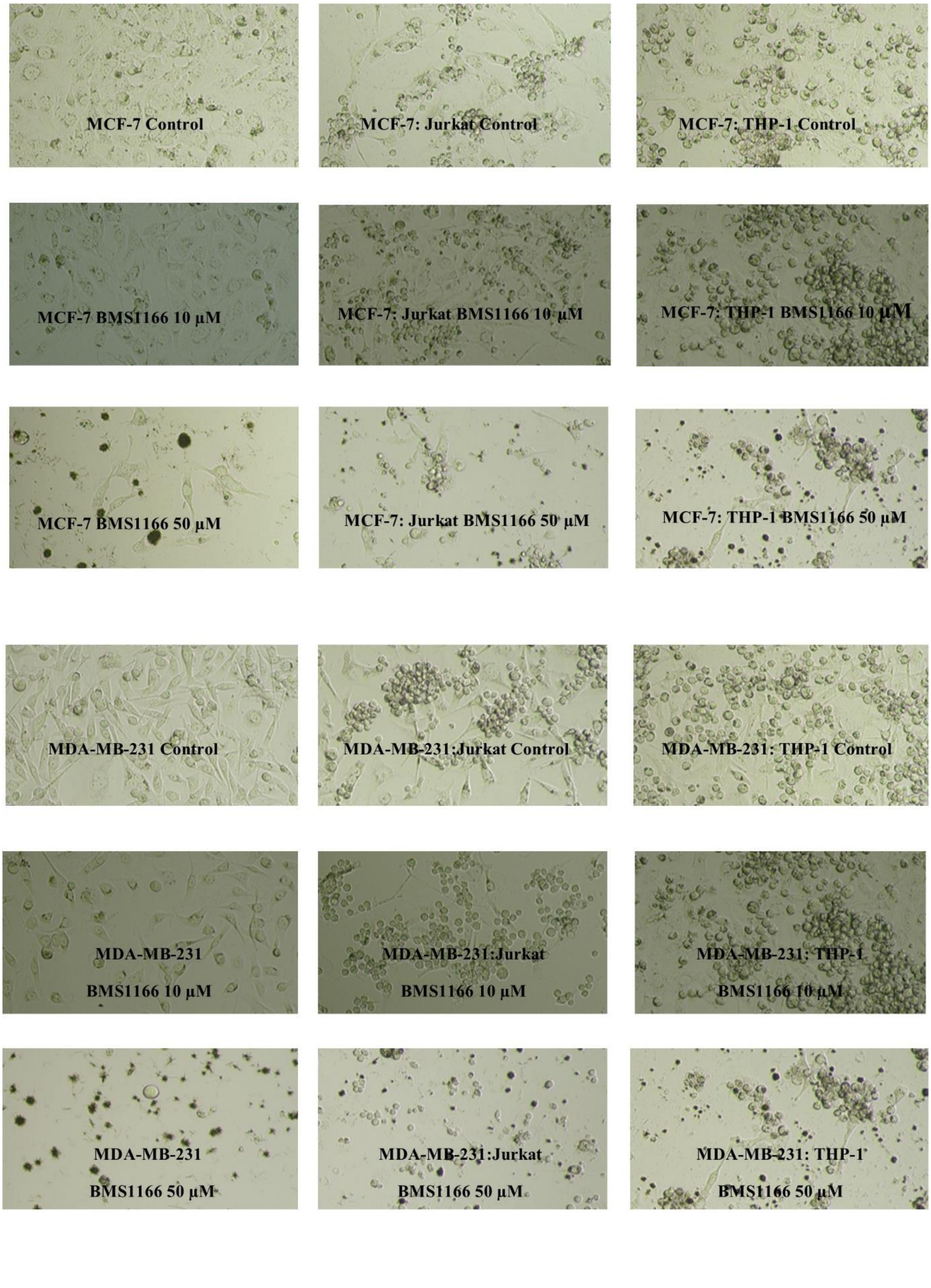

Figure S3. Cell viability of BMS1166-treated MCF-7 and MDA-MB-231 breast cancer cell lines and co-culture groups. Cell images captured using an inverted microscope with 10x magnification.

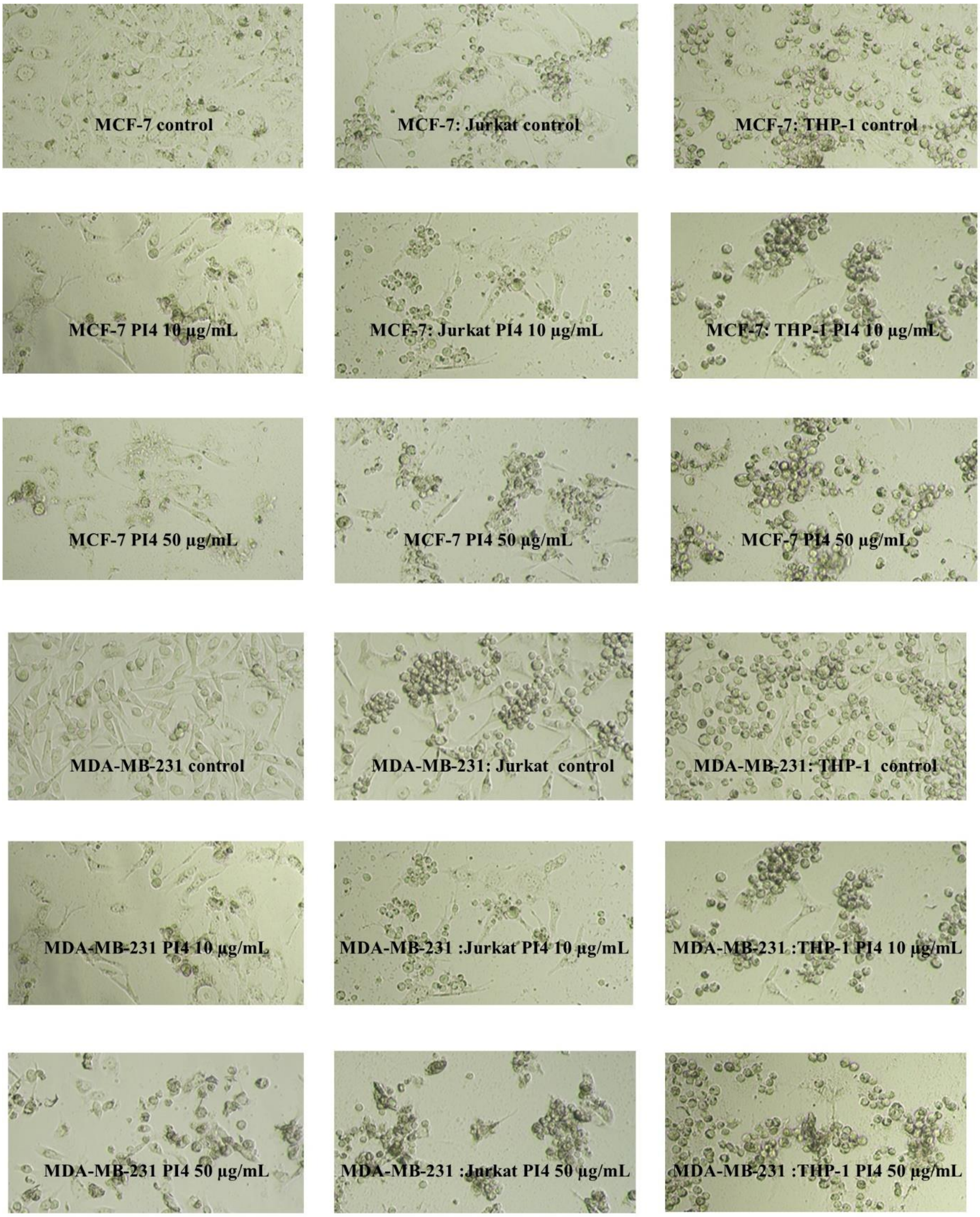

Figure S4. Cell viability of Human PD-L1 Inhibitor IV-treated MCF-7 and MDA-MB-231 breast cancer cell lines and co-culture groups. Cell images captured using an inverted microscope with 10x magnification.
